# The Association of Reported Experiences of Racial and Ethnic Discrimination in Health Care with COVID-19 Vaccination Status and Intent — United States, April 22, 2021–November 26, 2022

**DOI:** 10.15585/mmwr.mm7216a5

**Published:** 2023-04-21

**Authors:** Laurie D. Elam-Evans, Camara Phyllis Jones, Kushagra Vashist, David Yankey, Chalanda S. Smith, Jennifer L. Kriss, Peng-Jun Lu, Michael E. St. Louis, Noel T. Brewer, James A. Singleton

**Affiliations:** ^1^Immunization Services Division, National Center for Immunization and Respiratory Diseases, CDC; ^2^School of Global Affairs, King’s College London, London, England; ^3^Rollins School of Public Health, Emory University, Atlanta, Georgia; ^4^Division of Global HIV and TB, Center for Global Health, CDC; ^5^Department of Health Behavior, Gillings School of Global Public Health, University of North Carolina, Chapel Hill, North Carolina.

In 2021, the CDC Director declared that racism is a serious threat to public health,* reflecting a growing awareness of racism as a cause of health inequities, health disparities, and disease. Racial and ethnic disparities in COVID-19–related hospitalization and death (*1*,*2*) illustrate the need to examine root causes, including experiences of discrimination. This report describes the association between reported experiences of discrimination in U.S. health care settings and COVID-19 vaccination status and intent to be vaccinated by race and ethnicity during April 22, 2021–November 26, 2022, based on the analysis of interview data collected from 1,154,347 respondents to the National Immunization Survey–Adult COVID Module (NIS–ACM). Overall, 3.5% of adults aged ≥18 years reported having worse health care experiences compared with persons of other races and ethnicities (i.e., they experienced discrimination), with significantly higher percentages reported by persons who identified as non-Hispanic Black or African American (Black) (10.7%), non-Hispanic American Indian or Alaska Native (AI/AN) (7.2%), non-Hispanic multiple or other race (multiple or other race) (6.7%), Hispanic or Latino (Hispanic) (4.5%), non-Hispanic Native Hawaiian or other Pacific Islander (NHOPI) (3.9%), and non-Hispanic Asian (Asian) (2.8%) than by non-Hispanic White (White) persons (1.6%). Unadjusted differences in prevalence of being unvaccinated against COVID-19 among respondents reporting worse health care experiences than persons of other races and ethnicities compared with those who reported that their health care experiences were the same as those of persons of other races and ethnicities were statistically significant overall (5.3) and for NHOPI (19.2), White (10.5), multiple or other race (5.7), Black (4.6), Asian (4.3), and Hispanic (2.6) adults. Findings were similar for vaccination intent. Eliminating inequitable experiences in health care settings might help reduce some disparities in receipt of a COVID-19 vaccine.

NIS–ACM is a random-digit–dialed mobile telephone survey of adults aged ≥18 years in the 50 states, the District of Columbia, selected local areas, and selected U.S. territories.^†^ During the data collection period for this report, monthly survey response rates ranged from 17% to 23%. Reported experiences of racial and ethnic discrimination were assessed by the question, “When seeking health care in the last 2 years, do you feel your experiences were worse than, the same as, or better than (those of) persons of other races or ethnicities?” (hereafter worse, same, and better). “Don’t know” was also a valid response option. This question was adapted from CDC’s Behavioral Risk Factor Surveillance System (BRFSS) Reactions to Race module.^§^ The question was field tested, cognitively tested, and approved by the Office of Management and Budget, then slightly modified for NIS-ACM by adding ethnicities and expanding the time frame to 2 years because of COVID-19. Because of the interest in examining reported inequitable experiences in health care and the association of these experiences with COVID-19 vaccination behaviors, the focus for the analysis of discrimination was a comparison of worse experiences with same experiences. COVID-19 vaccination status^¶^ was assessed by three questions: “Have you received at least one dose of a COVID-19 vaccine?,” “Which brand of COVID-19 vaccine did you receive for your first dose?,” and “How many doses of a COVID-19 vaccine have you received?” The last two questions assessed completion of the primary series or partial vaccination. Among persons who had not received any COVID-19 vaccine doses, vaccination intent** was assessed by the question, “How likely are you to get a COVID-19 vaccine?” The outcomes of interest in this study were 1) being unvaccinated against COVID-19 and 2) among unvaccinated persons, definitely not intending to get vaccinated. Unadjusted and adjusted prevalence differences compared those reporting experiences that were worse than and the same as those of persons of other races and ethnicities. T-tests were used to assess statistical significance of unadjusted prevalence differences. Adjusted prevalence differences were based on multivariable logistic regression and predictive marginals assessing the association of reported discrimination with vaccination status and vaccination intent, overall and by race and ethnicity, controlling for age, sex, education, poverty status, metropolitan statistical area (urban, suburban, or rural residence), U.S. Census Bureau region, and health insurance status. Adjusted and unadjusted prevalence differences were considered statistically significant at p<0.05. Analyses were conducted for respondents overall and separately for AI/AN, Asian, Black, Hispanic, NHOPI, White, and multiple or other race respondents. Data were weighted to represent the noninstitutionalized U.S. adult population and to adjust for nonresponse and households without a telephone. Data were calibrated to state vaccine administration data reported to CDC.^††^ SAS (version 9.4; SAS Institute) and SUDAAN (version 11; RTI International) were used for statistical analyses. This activity was reviewed by CDC and was conducted consistent with applicable federal law and CDC policy.^§§^

Overall, 3.5% of respondents reported that their experiences in health care were worse than those of persons of other races and ethnicities (Supplementary Table; https://stacks.cdc.gov/view/cdc/127243) (Supplementary Figure 1; https://stacks.cdc.gov/view/cdc/127237). Significantly higher percentages of having worse experiences were reported by Black (10.7%), AI/AN (7.2%), multiple or other race (6.7%), Hispanic (4.5%), NHOPI (3.9%) and Asian (2.8%) adults than by White (1.6%) adults. The prevalence of being unvaccinated against COVID-19 was significantly higher among respondents reporting worse experiences in health care than among those reporting the same experiences, with unadjusted prevalence differences of 5.3 overall, and 19.2 among NHOPI, 10.5 among White, 5.7 among multiple and other race, 4.6 among Black, 4.3 among Asian, and 2.6 among Hispanic adults (Table 1) (Figure). After adjustment, the prevalence differences associated with being unvaccinated were attenuated but remained statistically significant overall (3.2) as well as for NHOPI (14.6), White (6.1), Black (2.7), Asian, (3.8), and Hispanic (3.0) adults (Table 1) (Supplementary Figure 2; https://stacks.cdc.gov/view/cdc/127242).

**TABLE 1:** Association between experience with racial and ethnic discrimination* when seeking health care and COVID-19 vaccination status, overall and by race and ethnicity — National Immunization Survey–Adult COVID Module, April 22, 2021–November 26, 2022

Race and ethnicity/ Experience	COVID–19 vaccination status								
	Not vaccinated			Partially vaccinated^†^			Completed primary vaccination series^§^		
	Unadjusted		Adjusted^¶^	Unadjusted		Adjusted^¶^	Unadjusted		Adjusted^¶^
	%, by row (95% CI)	PD (95% CI)	PD (95% CI)	%, by row (95% CI)	PD (95% CI)	PD (95% CI)	%, by row (95% CI)	PD (95% CI)	PD (95% CI)
**Overall**									
Worse	28.0 (27.0 to 29.0)	5.3 (4.3 to 6.3)**	3.2 (2.4 to 4.0)**	3.0 (2.7 to 3.4)	0.6 (0.2 to 0.9)**	0.3 (0 to 0.6)	68.6 (67.6 to 69.6)	–5.9 (–7.0 to –4.9)**	–3.5 (–4.4 to –2.6)**
Same	22.7 (22.5 to 22.9)	Ref	Ref	2.5 (2.4 to 2.5)	Ref	Ref	74.5 (74.3 to 74.8)	Ref	Ref
Better	8.6 (8.3 to 8.8)	–14.1 (–14.5 to –13.8)**	–10.4 (–10.8 to –10.1)**	1.6 (1.5 to 1.7)	–0.8 (–1.0 to –0.7)**	–0.5 (–0.6 to –0.3)**	89.5 (89.2 to 89.8)	15.0 (14.6 to 15.3)**	10.8 (10.4 to 11.2)**
Don't know	19.2 (18.9 to 19.6)	–3.4 (–3.9 to –3.0)**	–0.5 (–0.9 to –0.1)**	2.2 (2.0 to 2.3)	–0.3 (–0.5 to –0.2)**	–0.1 (–0.2 to 0.1)	78.1 (77.8 to 78.5)	3.6 (3.1 to 4.0)**	0.5 (0.1 to 0.9)**
American Indian or Alaska Native, non-Hispanic									
Worse	39.0 (32.5 to 45.6)	4.0 (–3.0 to 10.9)	3.0 (–3.1 to 9.2)	2.4 (1.0 to 3.8)	–0.6 (–2.2 to 1.0)	–0.8 (–2.5 to 0.8)	57.5 (51.1 to 63.9)	–4.0 (–10.9 to 2.8)	–2.8 (–8.8 to 3.2)
Same	35.1 (32.6 to 37.5)	Ref	Ref	3.1 (2.2 to 3.9)	Ref	Ref	61.5 (59.1 to 64.0)	Ref	Ref
Better	23.8 (19.9 to 27.7)	–11.2 (–15.8 to –6.7)**	–7.0 (–11.6 to –2.4)**	1.7 (0.8 to 2.6)	–1.3 (–2.5 to –0.1)**	–1.4 (–2.7 to –0.2)**	72.5 (68.2 to 76.9)	11.0 (6.0 to 16.0)**	6.8 (2.0 to 11.5)**
Don't know	36.6 (31.6 to 41.7)	1.6 (–4.0 to 7.2)	4.4 (–0.7 to 9.5)	2.3 (1.2 to 3.4)	–0.8 (–2.1 to 0.6)	–1.0 (–2.5 to 0.4)	59.7 (54.6 to 64.7)	–1.9 (–7.5 to 3.8)	–3.9 (–9.0 to 1.3)
Asian, non-Hispanic									
Worse	9.4 (5.3 to 13.4)	4.3 (0.2 to 8.4)**	3.8 (0 to 7.5)**	1.9 (0.6 to 3.2)	0.1 (–1.3 to 1.4)	–0.2 (–1.5 to 1.0)	88.7 (84.5 to 92.9)	–4.2 (–8.5 to 0)	–3.5 (–7.3 to 0.4)
Same	5.1 (4.6 to 5.7)	Ref	Ref	1.9 (1.6 to 2.1)	Ref	Ref	92.9 (92.3 to 93.5)	Ref	Ref
Better	4.9 (3.8 to 6.0)	–0.2 (–1.4 to 1.0)	0 (–1.3 to 1.2)	1.4 (1.0 to 1.9)	–0.4 (–1.0 to 0.1)	–0.4 (–1.0 to 0.1)	93.5 (92.4 to 94.7)	0.6 (–0.7 to 1.9)	0.4 (–0.9 to 1.8)
Don't know	5.4 (4.4 to 6.4)	0.3 (–0.9 to 1.5)	0.1 (–1.0 to 1.3)	1.9 (1.4 to 2.3)	0 (–0.5 to 0.5)	0.1 (–0.5 to 0.6)	92.2 (91.0 to 93.5)	–0.7 (–2.1 to 0.7)	–0.6 (–2.0 to 0.7)
Black, non-Hispanic									
Worse	25.3 (23.7 to 26.8)	4.6 (2.9 to 6.2)**	2.7 (1.3 to 4.1)**	3.2 (2.7 to 3.8)	0.3 (–0.3 to 0.9)	0.1 (–0.4 to 0.7)	71.3 (69.7 to 72.9)	–4.6 (–6.3 to –2.9)**	–2.7 (–4.2 to –1.3)**
Same	20.7 (20.1 to 21.3)	Ref	Ref	3.0 (2.7 to 3.2)	Ref	Ref	75.9 (75.3 to 76.5)	Ref	Ref
Better	19.4 (18.1 to 20.8)	–1.2 (–2.7 to 0.2)	1.3 (–0.2 to 2.7)	2.8 (2.3 to 3.2)	–0.2 (–0.7 to 0.3)	0.1 (–0.5 to 0.6)	77.2 (75.8 to 78.6)	1.3 (–0.3 to 2.8)	–1.2 (–2.7 to 0.3)
Don't know	22.6 (21.4 to 23.8)	1.9 (0.6 to 3.3)**	3.3 (2.0 to 4.6)**	3.1 (2.6 to 3.6)	0.1 (–0.5 to 0.6)	0.2 (–0.4 to 0.7)	73.5 (72.2 to 74.8)	–2.4 (–3.8 to –1.0)**	–3.7 (–5.0 to –2.4)**
Hispanic									
Worse	22.6 (20.5 to 24.7)	2.6 (0.4 to 4.8)**	3.0 (0.9 to 5.2)**	3.3 (2.4 to 4.2)	0.3 (–0.7 to 1.2)	0.3 (–0.7 to 1.2)	73.7 (71.5 to 75.9)	–2.9 (–5.2 to –0.6)**	–3.2 (–5.4 to –1.0)**
Same	20.0 (19.4 to 20.5)	Ref	Ref	3.1 (2.9 to 3.3)	Ref	Ref	76.6 (76.0 to 77.1)	Ref	Ref
Better	15.0 (14.0 to 16.0)	–5.0 (–6.1 to –3.9)**	–2.7 (–3.9 to –1.5)**	2.8 (2.4 to 3.2)	–0.3 (–0.7 to 0.2)	–0.2 (–0.7 to 0.3)	81.5 (80.5 to 82.5)	4.9 (3.8 to 6.1)**	2.6 (1.3 to 3.9)**
Don't know	21.8 (20.6 to 23.1)	1.8 (0.5 to 3.2)**	3.1 (1.7 to 4.5)**	3.5 (3.0 to 4.0)	0.4 (–0.2 to 1.0)	0.4 (–0.2 to 1.0)	74.1 (72.8 to 75.4)	–2.5 (–3.9 to –1.0)**	–3.6 (–5.1 to –2.2)**
Native Hawaiian or other Pacific Islander, non-Hispanic									
Worse	42.1 (29.1 to 55.1)^††^	19.2 (5.9 to 32.6)**^,††^	14.6 (2.0 to 27.1)**^,††^	2.0 (0.1 to 4.0)	–0.3 (–2.4 to 1.8)	–0.7 (–2.8 to 1.3)	55.3 (42.5 to 68.2)^††^	–19.1 (–32.3 to –5.8)^††^	–14.2 (–26.9 to –1.5)**^,††^
Same	22.9 (19.7 to 26.1)	Ref	Ref	2.3 (1.6 to 3.1)	Ref	Ref	74.4 (71.1 to 77.7)	Ref	Ref
Better	16.4 (11.0 to 21.8)	–6.5 (–12.8 to –0.2)**	–3.0 (–9.2 to 3.1)	2.1 (1.1 to 3.0)	–0.2 (–1.5 to 1.0)	0.1 (–1.3 to 1.5)	81.2 (75.7 to 86.7)	6.8 (0.4 to 13.2)**	3.0 (–3.1 to 9.2)
Don't know	19.9 (13.3 to 26.5)	–3.0 (–10.4 to 4.4)	0.6 (–6.1 to 7.4)	3.0 (1.0 to 5.1)	0.7 (–1.5 to 2.9)	0.9 (–1.4 to 3.1)	76.7 (69.9 to 83.5)	2.3 (–5.3 to 9.9)	–1.4 (–7.9 to 5.1)
White, non-Hispanic									
Worse	35.4 (33.5 to 37.3)	10.5 (8.7 to 12.4)**	6.1 (4.6 to 7.7)**	2.7 (2.1 to 3.3)	0.5 (–0.1 to 1.1)	0.1 (–0.4 to 0.6)	61.5 (59.6 to 63.4)	–11.2 (–13.1 to –9.3)**	–6.5 (–8.1 to –4.9)**
Same	24.9 (24.6 to 25.1)	Ref	Ref	2.2 (2.1 to 2.3)	Ref	Ref	72.7 (72.4 to 73.0)	Ref	Ref
Better	5.9 (5.6 to 6.2)	–19.0 (–19.3 to –18.6)**	–14.6 (–15.0 to –14.2)**	1.3 (1.2 to 1.4)	–0.9 (–1.1 to –0.8)**	–0.5 (–0.7 to –0.4)**	92.7 (92.4 to 92.9)	20.0 (19.6 to 20.4)**	15.1 (14.7 to 15.5)**
Don't know	18.4 (18.0 to 18.8)	–6.5 (–7.0 to –6.0)**	–2.9 (–3.4 to –2.5)**	1.8 (1.7 to 1.9)	–0.4 (–0.5 to –0.3)**	–0.2 (–0.3 to 0)**	79.4 (79.0 to 79.8)	6.8 (6.2 to 7.3)**	3.0 (2.6 to 3.5)**
Multiple or other, non-Hispanic									
Worse	36.3 (31.9 to 40.7)	5.7 (1.1 to 10.3)**	3.4 (–0.7 to 7.5)	3.5 (2.1 to 5.0)	0.9 (–0.5 to 2.4)	0.9 (–0.6 to 2.5)	59.8 (55.4 to 64.2)	–6.8 (–11.4 to –2.2)**	–4.4 (–8.6 to –0.3)**
Same	30.6 (29.3 to 31.9)	Ref	Ref	2.6 (2.2 to 3.1)	Ref	Ref	66.6 (65.2 to 67.9)	Ref	Ref
Better	14.6 (12.6 to 16.6)	–16.0 (–18.4 to –13.6)**	–11.1 (–13.6 to –8.6)**	2.1 (1.4 to 2.7)	–0.6 (–1.4 to 0.2)	–0.5 (–1.3 to 0.3)	83.0 (80.9 to 85.1)	16.4 (13.9 to 18.9)**	11.4 (8.8 to 14.0)**
Don't know	30.6 (28.2 to 33.0)	0 (–2.7 to 2.8)	2.6 (0 to 5.1)	2.5 (1.6 to 3.4)	–0.1 (–1.1 to 0.9)	0 (–1.0 to 1.1)	66.4 (64.0 to 68.8)	–0.2 (–3.0 to 2.6)	–2.7 (–5.3 to –0.1)**

**Abbreviations:** MSA = metropolitan statistical area; PD = prevalence difference; Ref = referent group.

* Reported experiences of racial and ethnic discrimination were assessed by the question, “When seeking health care in the last 2 years, do you feel your experiences were worse than, the same as, or better than (those of) persons of other races or ethnicities?” Persons who reported “worse” experiences were considered to have experienced discrimination.

† Received 1 dose of the Pfizer-BioNTech vaccine or 1 dose of the Moderna vaccine.

^§^ Received 2 doses of the Pfizer-BioNTech vaccine, 2 doses of the Moderna vaccine, 1 dose of the Janssen (Johnson & Johnson) vaccine, or 2 doses of the Novavax vaccine (starting with August 2022 data).

^¶^ Adjusted prevalence differences were those adjusted for age (18–29, 30–39, 40–49, 50–64, 65–74, or ≥75 years), sex (female or male), education (high school diploma or less, some college, college graduate, or advanced degree), poverty status (below poverty, at or above poverty and <$75,000 annually, or at or above poverty and ≥$75,000 annually), MSA status (MSA principal city, MSA nonprincipal city, or non-MSA), U. S. Census Bureau region (Northeast, Midwest, South, or West), and health insurance status (insured or not insured).

** Statistically significant difference compared with Ref (“same as”) (p<0.05).

^††^ 95% CI >20 might not be reliable.

**FIGURE:**
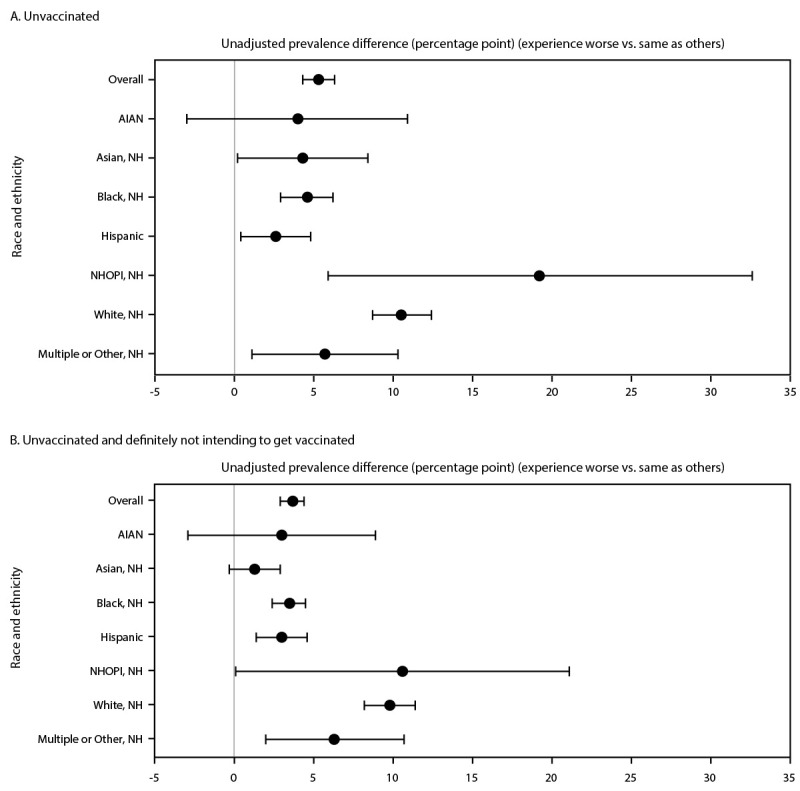
Unadjusted association* between experience with racial and ethnic discrimination^†^ while seeking health care and being (A) unvaccinated and (B) unvaccinated and definitely not intending to get vaccinated, overall and by race and ethnicity^§^— National Immunization Survey – Adult COVID Module, April 22, 2021–November 26, 2022 **Abbreviations**: AI/AN = American Indian or Alaska Native; NHOPI = Native Hawaiian or other Pacific Islander; NH = non-Hispanic * 95% CIs indicated by error bars; 95% CIs that exclude zero are statistically significant. ^†^ Reported experiences of racial and ethnic discrimination were assessed by the question, “When seeking health care in the last 2 years, do you feel your experiences were worse than, the same as, or better than (those of) persons of other races or ethnicities?” Persons who reported “worse” experiences were considered to have experienced discrimination. ^§^ Hispanic persons could be of any race.

Among unvaccinated respondents, the prevalence of those who stated that they were definitely not intending to get vaccinated was significantly higher among those who reported worse experiences in health care than among those who reported that their experiences were the same as those of persons of other races and ethnicities, with unadjusted prevalence differences of 3.7 overall, and 10.6 among NHOPI, 9.8 among White persons, 6.3 among multiple and other race, 3.5 among Black, and 3.0 among Hispanic (Table 2) (Figure). After adjustment, the prevalence differences were attenuated but remained statistically significant overall (2.9) and for White (6.4), multiple and other race (4.9), Black (2.6), and Hispanic (3.3) adults (Table 2) (Supplementary Figure 2; https://stacks.cdc.gov/view/cdc/127242).

**TABLE 2:** Association between experience with racial and ethnic discrimination* when seeking health care and COVID-19 vaccination intent, overall and by race and ethnicity — National Immunization Survey–Adult COVID Module, April 22, 2021–November 26, 2022

Race and ethnicity/ Experience	COVID–19 vaccination intent of unvaccinated respondents														
	Definitely will get vaccinated			Probably will get vaccinated			Unsure about getting vaccinated			Probably will not get vaccinated			Definitely will not get vaccinated		
	Unadjusted^†^		Adjusted^§^	Unadjusted^†^		Adjusted^§^	Unadjusted^†^		Adjusted^§^	Unadjusted^†^		Adjusted^§^	Unadjusted^†^		Adjusted^§^
	% (95% CI)	PD (95% CI)	PD (95% CI)	% (95% CI)	PD (95% CI)	PD (95% CI)	% (95% CI)	PD (95% CI)	PD (95% CI)	% (95% CI)	PD (95% CI)	PD (95% CI)	% (95% CI)	PD (95% CI)	PD (95% CI)
Overall															
Worse	2.5 (2.1 to 2.8)	0.6 (0.3 to 1.0)^¶^	0.3 (0 to 0.6)^¶^	2.7 (2.3 to 3.0)	0.1 (–0.2 to 0.5)	–0.1 (–0.4 to 0.2)	4.8 (4.3 to 5.3)	1.1 (0.6 to 1.5)^¶^	0.5 (0.1 to 0.9)^¶^	3.9 (3.5 to 4.4)	–0.2 (–0.7 to 0.2)	–0.3 (–0.7 to 0.1)	14.1 (13.4 to 14.9)	3.7 (2.9 to 4.4)^¶^	2.9 (2.2 to 3.5)^¶^
Same	1.8 (1.8 to 1.9)	Ref	Ref	2.5 (2.5 to 2.6)	Ref	Ref	3.7 (3.6 to 3.8)	Ref	Ref	4.2 (4.1 to 4.3)	Ref	Ref	10.4 (10.3 to 10.6)	Ref	Ref
Better	1.4 (1.3 to 1.5)	–0.4 (–0.6 to –0.3)^¶^	0 (–0.2 to 0.1)	1.4 (1.3 to 1.5)	–1.2 (–1.3 to –1.0)^¶^	–0.7 (–0.9 to –0.5)^¶^	1.7 (1.6 to 1.9)	–2.0 (–2.2 to –1.8)^¶^	–1.3 (–1.5 to –1.2)^¶^	1.3 (1.2 to 1.4)	–2.9 (–3.1 to –2.7)^¶^	–2.3 (–2.5 to –2.2)^¶^	2.8 (2.6 to 2.9)	–7.7 (–7.9 to –7.5)^¶^	–6.3 (–6.5 to –6.0)^¶^
Don't know	1.7 (1.5 to 1.8)	–0.2 (–0.3 to 0)^¶^	0.1 (–0.1 to 0.2)	1.9 (1.8 to 2.0)	–0.7 (–0.8 to –0.5)^¶^	–0.3 (–0.5 to –0.1)^¶^	3.5 (3.3 to 3.7)	–0.2 (–0.4 to –0)^¶^	0.3 (0.1 to 0.5)^¶^	3.1 (3.0 to 3.3)	–1.0 (–1.2 to –0.9)^¶^	–0.4 (–0.6 to –0.2)^¶^	9.1 (8.8 to 9.3)	–1.4 (–1.7 to –1.1)^¶^	–0.2 (–0.5 to 0.1)
American Indian or Alaska Native, non-Hispanic															
Worse	1.7 (0.4 to 2.9)	–1.0 (–2.5 to 0.6)	–1.1 (–2.5 to 0.4)	2.3 (0.8 to 3.8)	–0.4 (–2.2 to 1.4)	–0.2 (–2.0 to 1.6)	11.0 (5.1 to 16.9)	5.0 (–1.1 to 11.0)	4.7 (–0.4 to 9.8)	2.8 (1.2 to 4.4)	–2.9 (–4.9 to –0.9)^¶^	–2.8 (–4.7 to –1.0)^¶^	21.2 (15.6 to 26.7)	3.0 (–2.9 to 8.9)	2.2 (–3.4 to 7.9)
Same	2.6 (1.7 to 3.6)	Ref	Ref	2.7 (1.8 to 3.7)	Ref	Ref	6.0 (4.8 to 7.3)	Ref	Ref	5.7 (4.5 to 6.8)	Ref	Ref	18.2 (16.1 to 20.2)	Ref	Ref
Better	2.6 (1.2 to 3.9)	–0.1 (–1.7 to 1.5)	0.1 (–1.6 to 1.8)	1.6 (0.7 to 2.5)	–1.1 (–2.5 to 0.2)	–0.8 (–2.2 to 0.6)	4.6 (2.7 to 6.6)	–1.4 (–3.7 to 0.9)	–0.6 (–3.0 to 1.8)	2.8 (1.6 to 3.9)	–2.9 (–4.5 to –1.2)^¶^	–2.0 (–3.8 to –0.3)^¶^	12.3 (9.1 to 15.6)	–5.8 (–9.7 to– 2.0)^¶^	–4.1 (–8.1 to 0)^¶^
Don't know	1.7 (0.9 to 2.5)	–0.9 (–2.1 to 0.3)	–0.8 (–2.0 to 0.5)	3.8 (1.4 to 6.1)	1.0 (–1.5 to 3.6)	1.5 (–1.1 to 4.0)	7.7 (5.4 to 10.1)	1.7 (–0.9 to 4.3)	2.3 (–0.4 to 5.1)	6.9 (3.0 to 10.7)	1.2 (–2.8 to 5.2)	2.0 (–1.8 to 5.8)	16.9 (13.3 to 20.4)	–1.3 (–5.4 to 2.8)	–0.5 (–4.7 to 3.7)
Asian, non-Hispanic															
Worse	1.9 (–0.1 to 3.9)	0.5 (–1.5 to 2.5)	0.4 (–1.4 to 2.2)	0.8 (0.2 to 1.3)	–0.2 (–0.9 to 0.4)	–0.2 (–0.9 to 0.4)	3.4 (0.2 to 6.5)	2.4 (–0.8 to 5.5)	2.1 (–0.7 to 4.9)	1.2 (0.1 to 2.2)	0.4 (–0.7 to 1.5)	0.4 (–0.7 to 1.6)	2.2 (0.6 to 3.8)	1.3 (–0.3 to 2.9)	1.1 (–0.4 to 2.6)
Same	1.4 (1.1 to 1.7)	Ref	Ref	1.0 (0.8 to 1.2)	Ref	Ref	1.0 (0.8 to 1.3)	Ref	Ref	0.8 (0.5 to 1.1)	Ref	Ref	1.0 (0.7 to 1.2)	Ref	Ref
Better	1.6 (1.0 to 2.2)	0.2 (–0.5 to 0.9)	0.2 (–0.5 to 1.0)	1.1 (0.6 to 1.6)	0.1 (–0.5 to 0.7)	0.1 (–0.5 to 0.7)	0.8 (0.4 to 1.1)	–0.3 (–0.6 to 0.1)	–0.2 (–0.6 to 0.2)	1.0 (0.3 to 1.6)	0.2 (–0.5 to 0.9)	0.3 (–0.5 to 1.1)	0.5 (0.2 to 0.7)	–0.5 (–0.8 to –0.2)^¶^	–0.5 (–0.8 to –0.1)^¶^
Don't know	1.8 (1.1 to 2.6)	0.5 (–0.3 to 1.2)	0.4 (–0.4 to 1.2)	0.7 (0.3 to 1.0)	–0.4 (–0.7 to 0)	–0.4 (–0.8 to 0)	1.3 (0.8 to 1.8)	0.3 (–0.2 to 0.9)	0.4 (–0.2 to 0.9)	0.4 (0.2 to 0.6)	–0.4 (–0.7 to –0.1)^¶^	–0.4 (–0.7 to –0.1)^¶^	1.1 (0.6 to 1.6)	0.2 (–0.4 to 0.7)	0.1 (–0.4 to 0.6)
Black, non-Hispanic															
Worse	2.2 (1.8 to 2.6)	–0.4 (–0.8 to 0.1)	–0.4 (–0.9 to 0)	3.2 (2.6 to 3.9)	–0.5 (–1.2 to 0.2)	–0.8 (–1.4 to –0.2)^¶^	5.5 (4.7 to 6.4)	0.7 (–0.1 to 1.6)	0.4 (–0.4 to 1.2)	4.7 (3.7 to 5.6)	1.3 (0.3 to 2.3)^¶^	0.9 (0.1 to 1.8)^¶^	9.7 (8.7 to 10.7)	3.5 (2.4 to 4.5)^¶^	2.6 (1.6 to 3.6)^¶^
Same	2.6 (2.3 to 2.8)	Ref	Ref	3.8 (3.5 to 4.1)	Ref	Ref	4.8 (4.5 to 5.1)	Ref	Ref	3.3 (3.1 to 3.6)	Ref	Ref	6.2 (5.9 to 6.6)	Ref	Ref
Better	2.7 (2.2 to 3.3)	0.2 (–0.4 to 0.8)	0.3 (–0.3 to 0.9)	3.2 (2.6 to 3.9)	–0.5 (–1.2 to 0.2)	0 (–0.8 to 0.8)	4.7 (4.0 to 5.5)	–0.1 (–0.9 to 0.7)	0.4 (–0.4 to 1.3)	2.5 (1.9 to 3.0)	–0.9 (–1.5 to –0.3)^¶^	–0.4 (–1.1 to 0.3)	6.3 (5.5 to 7.1)	0.1 (–0.8 to 1.0)	1.0 (0 to 2.0)^¶^
Don't know	2.6 (2.1 to 3.0)	0 (–0.5 to 0.5)	0.2 (–0.4 to 0.7)	3.1 (2.6 to 3.6)	–0.7 (–1.2 to –0.1)^¶^	–0.5 (–1.0 to 0.1)	6.1 (5.4 to 6.8)	1.3 (0.6 to 2.1)^¶^	1.5 (0.7 to 2.3)^¶^	3.2 (2.7 to 3.7)	–0.1 (–0.7 to 0.4)	0.2 (–0.4 to 0.8)	7.6 (6.8 to 8.4)	1.4 (0.5 to 2.3)^¶^	1.9 (1.0 to 2.8)^¶^
Hispanic															
Worse	3.0 (2.2 to 3.8)	0.5 (–0.3 to 1.4)	0.5 (–0.3 to 1.4)	3.0 (2.2 to 3.9)	–0.4 (–1.3 to 0.5)	–0.4 (–1.3 to 0.5)	4.5 (3.5 to 5.5)	0.4 (–0.6 to 1.4)	0.5 (–0.6 to 1.6)	2.6 (1.8 to 3.3)	–0.9 (–1.7 to –0.1)^¶^	–0.7 (–1.6 to 0.1)	9.5 (8.0 to 11.1)	3.0 (1.4 to 4.6)^¶^	3.3 (1.6 to 5.0)^¶^
Same	2.4 (2.2 to 2.7)	Ref	Ref	3.5 (3.2 to 3.7)	Ref	Ref	4.1 (3.8 to 4.4)	Ref	Ref	3.4 (3.2 to 3.7)	Ref	Ref	6.5 (6.2 to 6.8)	Ref	Ref
Better	2.3 (1.9 to 2.7)	–0.1 (–0.6 to 0.3)	0.2 (–0.3 to 0.7)	2.9 (2.5 to 3.3)	–0.5 (–1.0 to 0)^¶^	–0.2 (–0.8 to 0.4)	3.7 (3.2 to 4.2)	–0.4 (–1.0 to 0.2)	0.1 (–0.6 to 0.8)	1.8 (1.4 to 2.2)	–1.6 (–2.1 to –1.2)^¶^	–1.3 (–1.8 to –0.7)^¶^	4.2 (3.6 to 4.8)	–2.3 (–3.0 to –1.7)^¶^	–1.6 (–2.3 to –0.8)^¶^
Don't know	2.4 (1.9 to 2.9)	0 (–0.6 to 0.5)	0.1 (–0.5 to 0.6)	3.3 (2.8 to 3.8)	–0.2 (–0.7 to 0.4)	0.1 (–0.6 to 0.8)	5.0 (4.3 to 5.6)	0.8 (0.1 to 1.5)^¶^	1.0 (0.2 to 1.7)^¶^	3.1 (2.6 to 3.5)	–0.4 (–0.9 to 0.2)	0 (–0.6 to 0.6)	7.9 (7.0 to 8.7)	1.4 (0.5 to 2.2)^¶^	1.8 (0.9 to 2.8)^¶^
Native Hawaiian or other Pacific Islander, non-Hispanic															
Worse	4.1 (0 to 8.3)	1.2 (–3.1 to 5.6)	0.2 (–3.9 to 4.2)	0.6 (–0.1 to 1.3)	–2.4 (–3.9 to –1.0)^¶^	–2.0 (–4.4 to 0.5)	8.4 (2.9 to 13.9)	4.0 (–1.7 to 9.6)	2.9 (–2.9 to 8.7)	9.4 (–2.9 to 21.6)^¶,^**	5.5 (–6.9 to 17.9)**	6.5 (–8.8 to 21.8)**	19.6 (9.4 to 29.9)^¶,^**	10.6 (0.1 to 21.1)^¶,^**	5.4 (–3.2 to 14.0)
Same	2.9 (1.5 to 4.3)	Ref	Ref	3.0 (1.8 to 4.3)	Ref	Ref	4.4 (3.0 to 5.8)	Ref	Ref	3.9 (2.4 to 5.3)	Ref	Ref	9.0 (6.7 to 11.4)	Ref	Ref
Better	0.6 (0.1 to 1.1)	–2.3 (–3.8 to –0.8)^¶^	–2.4 (–3.9 to –0.8)^¶^	1.0 (0.3 to 1.7)	–2.1 (–3.5 to –0.6)^¶^	–1.1 (–3.2 to 0.9)	6.1 (1.9 to 10.4)	1.7 (–2.8 to 6.2)	2.5 (–1.9 to 6.8)	1.6 (0.7 to 2.4)	–2.3 (–3.9 to –0.7)^¶^	–2.1 (–3.7 to –0.5)^¶^	7.2 (3.9 to 10.6)	–1.8 (–5.9 to 2.3)	–0.6 (–5.1 to 3.9)
Don't know	4.7 (1.0 to 8.5)	1.9 (–2.2 to 5.9)	2.6 (–1.8 to 6.9)	0.7 (0.2 to 1.1)	–2.4 (–3.7 to –1.0)^¶^	–1.8 (–3.7 to 0.1)	2.9 (1.7 to 4.1)	–1.5 (–3.4 to 0.3)	–1.1 (–3.3 to 1.0)	6.0 (0.7 to 11.4)	2.2 (–3.3 to 7.7)	3.3 (–2.1 to 8.7)	5.8 (3.2 to 8.3)	–3.3 (–6.8 to 0.2)	–2.8 (–6.1 to 0.6)
White, non-Hispanic															
Worse	2.3 (1.6 to 3.1)	0.9 (0.1 to 1.7)^¶^	0.5 (–0.1 to 1.1)	2.1 (1.6 to 2.6)	0 (–0.5 to 0.6)	–0.1 (–0.5 to 0.4)	3.9 (3.1 to 4.7)	0.4 (–0.5 to 1.2)	–0.3 (–0.9 to 0.4)	4.1 (3.4 to 4.8)	–0.7 (–1.4 to 0)^¶^	–0.8 (–1.5 to –0.2)^§^	22.9 (21.3 to 24.4)	9.8 (8.2 to 11.4)^¶^	6.4 (5.0 to 7.7)^¶^
Same	1.4 (1.4 to 1.5)	Ref	Ref	2.1 (2.0 to 2.2)	Ref	Ref	3.5 (3.4 to 3.7)	Ref	Ref	4.8 (4.7 to 5.0)	Ref	Ref	13.1 (12.9 to 13.3)	Ref	Ref
Better	1.0 (0.9 to 1.2)	–0.4 (–0.5 to –0.3)^¶^	0 (–0.2 to 0.1)	0.9 (0.8 to 1.0)	–1.2 (–1.3 to –1.0)^¶^	–0.8 (–1.0 to –0.6)^¶^	1.0 (0.9 to 1.1)	to 2.5 (–2.7 to –2.4)^¶^	–2.0 (–2.1 to –1.8)^¶^	1.0 (0.9 to 1.1)	–3.8 (–4.0 to –3.6)^¶^	–3.1 (–3.3 to –2.9)^¶^	2.0 (1.8 to 2.1)	–11.1 (–11.4 to –10.8)^¶^	–9.1 (–9.4 to –8.8)^¶^
Don't know	1.3 (1.2 to 1.4)	–0.1 (–0.3 to 0)	0.1 (0 to 0.3)	1.5 (1.4 to 1.6)	–0.6 (–0.7 to –0.4)^¶^	–0.3 (–0.4 to –0.1)^¶^	2.9 (2.7 to 3.1)	–0.7 (–0.9 to –0.4)^¶^	–0.1 (–0.4 to 0.1)	3.2 (3.0 to 3.4)	–1.6 (–1.9 to –1.4)^¶^	–0.9 (–1.1 to –0.7)^¶^	9.6 (9.2 to 9.9)	–3.5 (–3.9 to –3.1)^¶^	–1.8 (–2.2 to –1.5)^¶^
Multiple or other, non-Hispanic															
Worse	3.4 (1.7 to 5.1)	0.8 (–0.9 to 2.6)	0.7 (–1.0 to 2.4)	2.0 (1.1 to 2.8)	–1.6 (–2.6 to –0.6)^¶^	–1.7 (–2.6 to –0.7)^¶^	4.0 (2.7 to 5.4)	–0.4 (–1.9 to 1.1)	–0.7 (–2.1 to 0.7)	5.7 (3.9 to 7.4)	0.4 (–1.5 to 2.3)	0.3 (–1.5 to 2.2)	21.2 (17.0 to 25.5)	6.3 (2.0 to 10.7)^¶^	4.9 (1.0 to 8.7)^¶^
Same	2.6 (2.0 to 3.1)	Ref	Ref	3.5 (2.9 to 4.1)	Ref	Ref	4.4 (3.8 to 5.0)	Ref	Ref	5.3 (4.6 to 5.9)	Ref	Ref	14.9 (13.9 to 15.9)	Ref	Ref
Better	2.0 (1.0 to 3.0)	–0.6 (–1.7 to 0.6)	0 (–1.4 to 1.4)	1.7 (1.0 to 2.4)	–1.8 (–2.7 to –0.9)^¶^	–1.2 (–2.2 to –0.1)^¶^	2.4 (1.5 to 3.3)	–2.0 (–3.1 to –1.0)^¶^	–1.4 (–2.7 to –0.2)^¶^	2.2 (1.4 to 3.1)	–3.0 (–4.1 to –2.0)^¶^	–2.1 (–3.3 to –0.8)^¶^	6.2 (4.9 to 7.4)	–8.7 (–10.3 to –7.1)^¶^	–7.0 (–8.7 to –5.4)^¶^
Don't know	2.8 (1.8 to 3.8)	0.3 (–0.9 to 1.4)	0.6 (–0.7 to 1.9)	2.5 (1.6 to 3.3)	–1.1 (–2.1 to –0)^¶^	–0.6 (–1.7 to 0.5)	5.6 (4.3 to 6.9)	1.2 (–0.2 to 2.6)	1.8 (0.3 to 3.3)^¶^	4.4 (3.4 to 5.4)	–0.9 (–2.1 to 0.3)	–0.4 (–1.7 to 0.8)	15.3 (13.5 to 17.2)	0.5 (–1.6 to 2.5)	1.0 (–1.0 to 3.0)

**Abbreviations**: MSA = metropolitan statistical area; PD = prevalence difference; Ref = referent group.

* Reported experiences of racial and ethnic discrimination were assessed by the question, “When seeking health care in the last 2 years, do you feel your experiences were worse than, the same as, or better than (those of) persons of other races or ethnicities?” Persons who reported “worse” experiences were considered to have experienced discrimination.

^†^ The unadjusted row percentages for the five categories of vaccination intent add to the unadjusted percentages of unvaccinated adults.

^§^ Adjusted prevalence differences were those adjusted for age (18–29, 30–39, 40–49, 50–64, 65–74, or ≥75 years), sex (female or male), education (high school diploma or less, some college, college graduate, or advanced degree), poverty status (below poverty, at or above poverty and <$75,000 annually, or at or above poverty and ≥$75,000 annually), MSA status (MSA principal city, MSA nonprincipal city, or non–MSA), U.S. Census Bureau region (Northeast, Midwest, South, or West), and health insurance status (insured or not insured).

^¶^ Statistically significant difference compared with Ref (“same as”) (p<0.05).

** 95% CI >20 might not be reliable.

## Discussion

Reported experiences of racial and ethnic discrimination in health care settings were associated with a difference in prevalence of nonvaccination against COVID-19 ranging from 2.6 (Hispanic adults) to 19.2 (NHOPI adults). Among unvaccinated adults, experiencing discrimination in health care settings was associated with a prevalence difference ranging from 3.0 (Hispanic adults) to 10.6 (NHOPI adults) for respondents who were definitely not intending to get vaccinated. Other studies have attributed disparities in COVID-19 vaccination to lower confidence in vaccination and more barriers to access (*3*,*4*). Few studies, however, have examined the association between reported experiences of discrimination in health care with COVID-19 vaccination status and intent to be vaccinated. One national survey^¶¶^ found that U.S. adults who reported experiences of discrimination in health care had lower COVID-19 vaccination coverage. A study on influenza vaccination identified similar findings. Specifically, seasonal influenza vaccination coverage among chronically ill U.S. adults who reported experiencing discrimination in health care was one half that of those who did not report these experiences (32% versus 60%; p = 0.009) (*5*). Addressing experiences of discrimination in health care settings might facilitate preventive care use, including COVID-19 vaccination.

The findings in this report are subject to at least eight limitations. First, response rates for the NIS–ACM were approximately 20%; therefore, bias might occur from household nonresponse and phoneless households. Data weighting might have partially mitigated this bias. Second, COVID-19 vaccination status was self-reported and subject to misclassification because of errors in recall or social desirability; however, given the recency of COVID-19 vaccine authorization and high awareness of the pandemic, errors in vaccination recall were likely minimal. Third, this report focuses on reported discrimination in health care settings during the previous 2 years. If respondents refrained from seeking health care because of the pandemic, true rates of discrimination could be higher or lower; CDC was unaware of any data to evaluate this potential limitation. Fourth, this survey did not sample institutionalized adults, including those experiencing incarceration or living in nursing homes, which might underestimate the impact of discrimination. Fifth, the adjusted prevalence differences might be biased because of misspecification of the multivariable models. Specifically, some adjustment variables, such as socioeconomic status and health insurance status, might not truly be independent of discrimination, but instead be linked in complex ways with race and ethnicity in these experiences. Sixth, there might be some uncontrolled covariates contributing to the observed association between reported racial and ethnic discrimination and COVID-19 vaccination status or intent. Seventh, the sample size for NHOPI was small, which might have yielded unreliable estimates. However, including NHOPI permitted analysis of as many racial and ethnic groups as possible. Finally, this is a cross-sectional study; therefore, causal inferences about the impact of reported discrimination on COVID-19 vaccination status and intent cannot be made.

Reported racial and ethnic discrimination appears to be associated with at least some disparities in COVID-19 vaccine receipt; thus, eliminating inequities in health care experiences might reduce some of this disparity and potentially increase vaccination coverage among adults. Some progress was made to address disparities early in the pandemic (*6*). To advance vaccine equity, CDC has funded national, state, local, and community-level partners*** who work with their communities to increase vaccine access, confidence, demand, and equity by training trusted messengers, setting up vaccination clinics, and conducting community outreach in local languages and dialects.^†††^^,^^§§§^

Additional studies and action are needed to understand and eliminate discrimination. CDC supported the examination of discrimination in 27 states to administer the Reactions to Race module on the 2022 and 2023 BRFSS, and analysis of experiences with racism is a part of the current research agenda (Personal communication, Machell Town, CDC, January 6, 2023), including assessment of structural racism (*7*). Prevention programs and prevention partners (i.e., community health workers and trusted messengers) could plan ways to address health care discrimination as a source of vaccine hesitancy when implementing health strategies (*8*). Health care providers might foster patient trust and improve adherence to recommended health interventions by becoming aware of patients’ potential negative health care experiences and of known incidents of historical mistreatment, and incorporating this sensitivity into their patient interactions. Since the CDC Director’s declaration that racism is a serious public health threat, CDC’s scientific research to address health inequities rooted in racism has expanded across the agency with a renewed commitment to better understanding both the social determinants of health (including poverty) and the social determinants of equity (including racism) (*9*) and to addressing the racial and ethnic health inequities revealed throughout the COVID-19 pandemic.^¶¶¶^

**Summary**

**What is already known about this topic?**

There is a growing awareness of racism as a cause of health inequities, health disparities, and disease.

**What is added by this report?**

Adults reporting experiences of racial and ethnic discrimination in health care had a significantly higher prevalence of being unvaccinated against COVID-19 overall and among most racial and ethnic groups.

**What are the implications for public health practice?**

Strategies to address inequitable experiences (discrimination) include increasing awareness by health care providers of patients’ potential negative health care experiences and known historical mistreatment and incorporating this sensitivity into their patient interactions. This action might foster patient trust, improve adherence to recommended health interventions, and reduce some COVID-19–related health disparities.

## Footnotes

*
https://www.cdc.gov/media/releases/2021/s0408-racism-health.html

† Local areas that received federal immunization funds under Section 317 of the Public Health Service Act are sampled separately in the National Immunization Survey. Local areas include Bexar County, Texas; Chicago, Illinois; Houston, Texas; New York, New York; and Philadelphia County, Pennsylvania. Three U.S. territories were sampled separately: Guam (April–July 2021 and April–July 2022), Puerto Rico, and U.S. Virgin Islands (January 2021–December 2021).

§ The question was one of six included in the Reactions to Race module that was piloted in 2002 and included as an optional module during 2002–2014. https://www.cdc.gov/brfss/questionnaires/index.htm

¶ Vaccination status was categorized as follows: not vaccinated/unvaccinated (zero doses), partially vaccinated (1 dose Pfizer-BioNTech or 1 dose Moderna), and fully vaccinated/completed a vaccine primary series (2 doses of the Pfizer-BioNTech vaccine, 2 doses of the Moderna vaccine, 2 doses of the Novavax vaccine [starting with August 2022 data], or 1 dose of the Janssen [Johnson & Johnson] vaccine). Immunocompromise status was also used to determine completion of the primary series.

** Vaccination intent was asked of respondents reporting they had not received any COVID-19 vaccinations and was categorized as follows: unvaccinated but definitely intend to get vaccinated, unvaccinated but probably will get vaccinated, unvaccinated and unsure about getting vaccinated, unvaccinated and probably will not get vaccinated, and unvaccinated and definitely will not get vaccinated.

†† Survey weights were calibrated to the COVID-19 vaccine administration data by jurisdiction, age group, and sex. https://covid.cdc.gov/covid-data-tracker/ (Accessed September 23, 2022).

§§ 45 C.F.R. part 46.102(l)(2); 21 C.F.R. part 56; 42 U.S.C. Sect. 241(d); 5 U.S.C. Sect. 552a; 44 U.S.C. Sect. 3501 et seq.

¶¶
https://www.brookings.edu/blog/how-we-rise/2021/10/20/discrimination-in-the-healthcare-system-is-leading-to-vaccination-hesitancy/

***
https://www.cdc.gov/media/releases/2021/p0317-COVID-19-Health-Disparities.html

†††
https://www.cdc.gov/media/releases/2021/p0325-community-healthworkers-support.html

§§§
https://www.cdc.gov/vaccines/health-equity/index.html

¶¶¶
https://www.cdc.gov/minorityhealth/racism-disparities/cdc-efforts.html
